# Walking-adaptability therapy after stroke: results of a randomized controlled trial

**DOI:** 10.1186/s13063-021-05742-3

**Published:** 2021-12-15

**Authors:** C. Timmermans, M. Roerdink, C. G. M. Meskers, P. J. Beek, T. W. J. Janssen

**Affiliations:** 1 Rehabilitation Research Center, Amsterdam, Overtoom 283, 1054 HW Amsterdam, The Netherlands; 2 Department of Human Movement Sciences, Faculty of Behavioural and Movement Sciences, Vrije Universiteit Amsterdam, Amsterdam Movement Sciences, Amsterdam, The Netherlands; 3 Department of Rehabilitation Medicine, Vrije Universiteit Amsterdam, Amsterdam Movement Sciences, Amsterdam, The Netherlands

**Keywords:** Rehabilitation, Stroke, Therapy, Gait, Walking speed, Walking adaptability

## Abstract

**Background:**

The ability to adapt walking to environmental properties and hazards, a prerequisite for safe ambulation, is often impaired in persons after stroke.

**Research question:**

The aim of this study was to compare the efficacy of two walking-adaptability interventions: a novel treadmill-based C-Mill therapy (using gait-dependent augmented reality) and the standard overground FALLS program (using physical context). We expected sustained improvements for both treatment groups combined but hypothesized better outcomes for C-Mill therapy than the FALLS program due to its expected greater amount of walking practice.

**Methods:**

In this pre-registered single-centre parallel group randomized controlled trial, forty persons after stroke (≥ 3 months ago) with walking and/or balance deficits were randomly allocated to either 5 weeks of C-Mill therapy or the FALLS program. The primary outcome measure was the standard walking speed as determined with the 10-meter walking test (10MWT). Additionally, context-specific walking speed was assessed in environments enriched with either stationary physical context (10MWT context) or suddenly appearing visual images (Interactive Walkway obstacles). The walking-adaptability scores of those enriched walking tests served as secondary outcome measures. Furthermore, a cognitive task was added to all three assessments to evaluate dual-task performance in this context. Finally, the participants’ experience and amount of walking practice were scored. The outcome measures were assessed at four test moments: pre-intervention (T0), post-intervention (T1), 5-week post-intervention retention (T2), and 1-year post-intervention follow-up (T3).

**Results:**

No significant group differences were found between the interventions for the primary outcome measure standard walking speed, but we found a greater improvement in context-specific walking speed with stationary physical context of the C-Mill therapy compared to the FALLS program at the post-intervention test, which was no longer significant at retention. Both interventions were well received, but C-Mill therapy scored better on perceived increased fitness than the FALLS program. C-Mill therapy resulted in twice as many steps per session of equal duration than the FALLS program. The “change-over-time” analyses for participants of both interventions combined showed no significant improvements in the standard walking speed; however, significant improvements were found for context-specific walking speed, walking adaptability, and cognitive dual-task performance.

**Significance:**

This study showed no between-group differences between the novel treadmill-based C-Mill therapy and the standard overground FALLS program with respect to the primary outcome measure standard walking speed. However, the greater amount of walking practice observed for the C-Mill group, an essential aspect of effective intervention programs after stroke, may underlie the reported increased perceived fitness and observed increased context-specific walking speed for the C-Mill group directly after the intervention. Although the “change-over-time” results for all participants combined showed no improvement in the standard walking speed, context-specific walking speed and walking adaptability showed sustained improvements after the interventions, underscoring the importance of including walking-adaptability training and assessment in rehabilitation post stroke.

**Trial registration:**

The Netherlands Trial Register NTR4030. Registered 11 June 2013.

**Supplementary Information:**

The online version contains supplementary material available at 10.1186/s13063-021-05742-3.

## Background

Walking in everyday life requires the ability to adapt walking to environmental circumstances and hazards [1]. This adaptability is often impaired in people after stroke [2, 3], which might contribute to the high risk of falling in this population [4, 5]. Moreover, attentional demands of walking are often elevated in people after stroke [6, 7], especially when step adjustments or step adjustments under time pressure are required [3, 8]. To improve safe community ambulation in people after stroke, practicing walking adaptability seems essential.

Several training programs have been developed to practice walking adaptability. For instance, the FALLS program [9] is an overground walking-adaptability training that involves practicing complex situations of community walking, such as walking over an obstacle course. The FALLS program is based on the Nijmegen Falls Prevention Program, which was designed for community-dwelling older adults with a history of falling and has been proven effective in reducing the number of falls in this population [10, 11]. The FALLS program (or a similar obstacle-course training) is the standard walking-adaptability intervention of choice in many Dutch rehabilitation centers. Treadmill-based therapy with augmented reality (C-Mill) is a novel and promising form of walking-adaptability training [8, 12–15]. The C-Mill is an instrumented treadmill equipped with a projector (Motek, Amsterdam, the Netherlands) to project visual images representing stepping targets or obstacles onto the walking surface. It has an embedded force platform that allows for gait-event detection and the provision of real-time feedback on performance [16]. The instrumented C-Mill further supports projection of interactive context in a gait-dependent manner, facilitating practicing step adjustments under high time-pressure demands, which is especially difficult for people after stroke [3, 8].

**Fig. 1:**
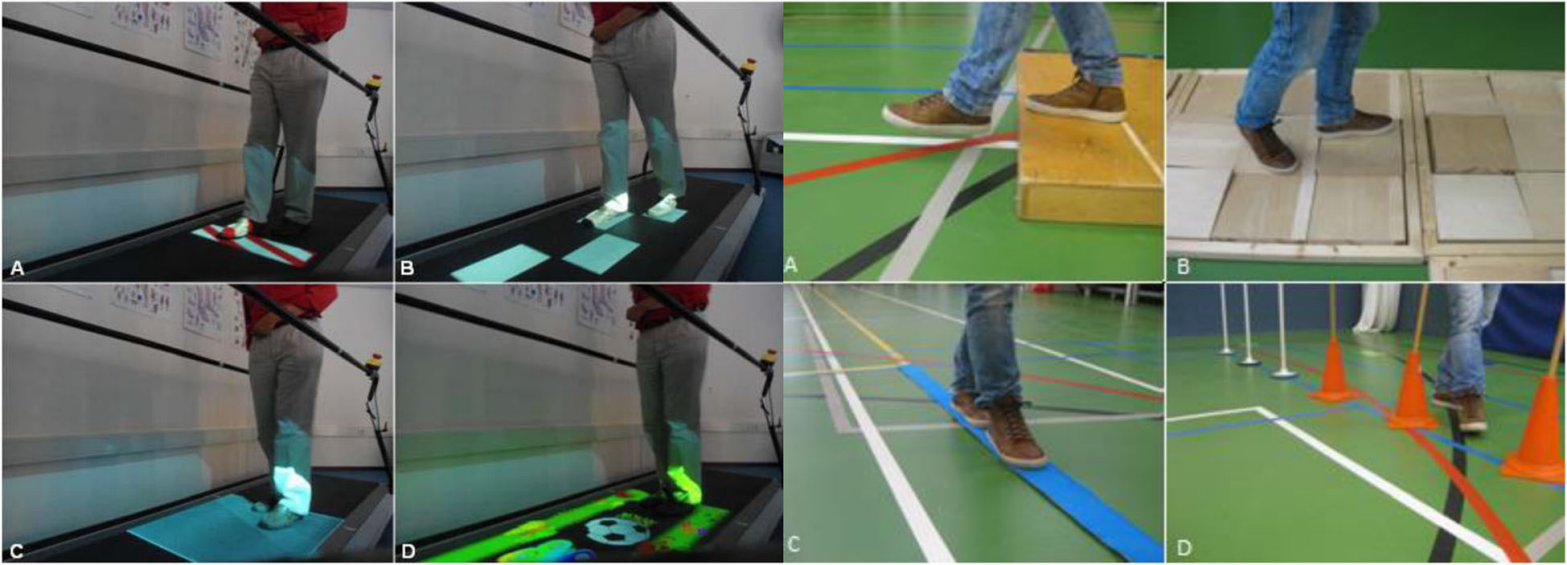
Snapshots of the two interventions aimed at improving walking adaptability. (Left) Walking-adaptability exercises of treadmill-based C-Mill therapy (CT); **A** obstacle avoidance, **B** goal-directed stepping, **C** gait acceleration and deceleration, and **D** a functional and interactive walking-adaptability game. (Right) Obstacle course of the overground FALLS program (FP); **A** obstacle avoidance, **B** walking over uneven terrain, **C** tandem walking, and **D** slalom walking

Both FALLS and C-Mill interventions (Fig. 1) comprise key ingredients for effective walking rehabilitation and motor learning (task and context specificity, variability in practice, and feedback of performance) [17–20]. However, the interventions differ in the way in which these ingredients are implemented. The FALLS program uses stationary physical context (e.g., real obstacles) to enrich the environment, whereas C-Mill therapy uses gait-dependent augmented reality (e.g., suddenly appearing projected obstacles attuned to the participant’s future foot placement). Moreover, the FALLS program entails overground gait training while C-Mill therapy offers treadmill-based gait training. Treadmill gait training has been suggested to elicit more steps per session of equal duration compared to overground gait training [13, 19]. Therefore, C-Mill therapy is likely to result in a higher amount of walking practice than the overground FALLS program (operationalized as the number of steps taken per unit session time).

The aim of the present study was to compare the efficacy of the novel treadmill-based C-Mill therapy (CT) with the standard overground FALLS program (FP) in persons after stroke in the Netherlands. Although we expected sustained improvements for both interventions combined (“change-over-time” analyses), it was hypothesized that CT results in better outcomes than the FP due to its expected higher amount of walking practice per session of equal duration (“between-group” analyses). To test this hypothesis, we compared the total number of steps as well as the number of adaptive steps (i.e., when context was provided) taken per training session and evaluated (differences in) immediate and long-term training effects of the interventions.

## Methods

### Study design

This was a pre-registered single-centre parallel group randomized controlled trial with pre-intervention (T0), post-intervention (T1), 5-week post-intervention retention (T2), and 1-year post-intervention follow-up (T3) tests. Participants were randomly assigned to either 5 weeks of CT or FP.

### Participants

Participants were recruited from the outpatient population of rehabilitation center Reade (Amsterdam, the Netherlands). Inclusion criteria were first-ever ischemic stroke ≥ 3 months before study entrance, Functional Ambulation Categories (FAC) score ≥ 4, hemiparesis and walking and/or balance deficits established by a physician. Exclusion criteria were orthopedic and other neurological disorders that affect walking (e.g., Parkinson’s disease), other treatments that could influence the effects of the interventions (e.g., recent Botulin toxin treatment of the lower extremity), contra-indication to physical activity (e.g., heart failure, severe osteoporosis), moderate or severe cognitive impairments as indicated by a Mini-Mental State Examination score below 21, severe uncorrected visual deficits, or inability to understand and execute simple instructions [21]. All participants provided written informed consent before the start of the trial. The protocol for the study was approved by the Medical Ethical Reviewing Committee of the VU University Medical Centre (Amsterdam, the Netherlands; protocol number 2013/53 and the Central Committee on Research Involving Human Subjects, CCMO, protocol number NL 42461.029.13). Serious adverse events (SAES) and adverse events (AES) were monitored during this trial.

### Sample size

The study of Yang et al. [6] allowed for a sample size calculation for post hoc analyses for significant group effects on walking speed with independent *t* tests. We aimed for a relative, clinically relevant, improvement in walking speed of 0.50 km/h (*∆*) with a common standard deviation (*SD*) of 0.47 km/h. The sample size calculation that was carried out resulted in a sample size of 14 participants in each group to achieve 80% power with a two-tailed *α* of 0.05 [21]. Considering a drop out of 10–25%, we decided to recruit 20 participants in each group, resulting in a total of 40 participants

### Randomization and blinding

After having provided informed consent, participants were randomly assigned to one of the two interventions using an automated, custom-made minimization algorithm written in MATLAB. This minimization of group differences used time after stroke, age, and FAC score as stratifying factors, which collectively determined 80% of group allocation. Due to the nature of the intervention, the assessors, physical therapists, and participants were not blinded to group allocation.

### Interventions: treadmill-based C-Mill therapy (CT) and overground FALLS program (FP)

CT is a treadmill-based training with a specific emphasis on practicing walking adaptability, using gait-dependent projector-generated context on the instrumented treadmill surface to elicit step adjustments. CT encompasses various exercises to practice avoidance of projected visual obstacles, foot positioning on a step-to-step basis to regular or irregular sequences of visual stepping targets (goal-directed stepping) with or without obstacles, gait acceleration, and deceleration by maintaining position within a projected walking area that moves along the treadmill, walking with tandem steps, and an interactive walking-adaptability game [8, 12]. C-Mill therapy is a patient-tailored type of training in that the therapist can adjust the difficulty of the different exercises by manipulating content parameters such as the obstacle size or available response time for obstacle negotiation. Therapists were encouraged to increase the level of difficulty as tolerated by the participant by either changing content parameters or increasing the treadmill belt speed.

FP is an overground walking therapy program aimed at reducing the number of falls in people after stroke by including walking-adaptability exercises. The program incorporates an obstacle course consisting of exercises to practice obstacle avoidance, foot positioning while walking over uneven terrain, tandem walking, and slalom walking. Therapists in this program are encouraged to increase the level of difficulty by adding cognitive and motor dual-tasks or to use visual constraints, as described in the predefined training protocol [9]. The program also incorporates exercises to simulate walking in a crowded environment and to practice falling techniques (one session per week).

Both interventions were matched in therapy session duration (90 min) and frequency (twice per week). CT group trained in pairs of two participants and the FP group trained in groups of 4–6 participants. Participants in both groups alternately trained and rested and received similar therapist attention (mean participant-to-therapist ratio, 2:1). Further details of the interventions can be found in the study protocol [21]. Participants successfully completed the intervention if they completed at least 7 out of the 10 training sessions.

### Procedure and set-ups

**Fig. 2:**
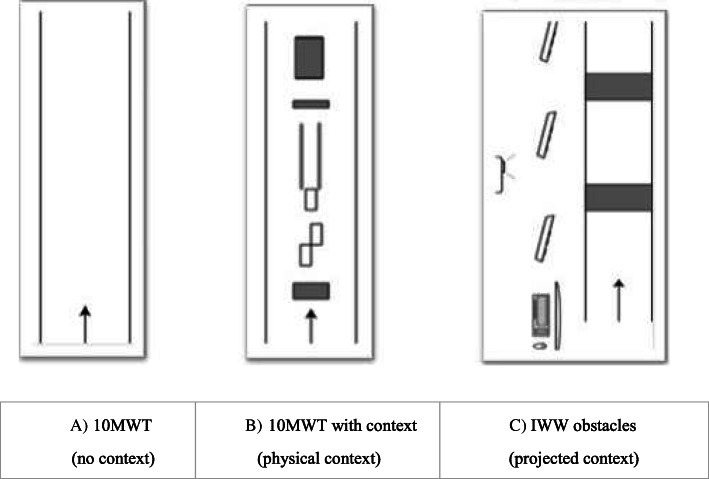
Schematic representations of the three walking assessments and the related outcome measures; **A** standard 10MWT, **B** 10MWT with physical context, comprising three obstacles (at 2.0 m, 7.5 m, and 9.0 m, of length × width × height 9.0 × 20.0 × 4.5 cm, 4.5 × 20.0 × 9.0 cm and 33.0 × 21.0 × 11.5 cm, respectively), a 2-m tandem-walking path (with a width of 20 cm) and three stepping targets (participants’ shoe length + 4 cm by shoe width + 4 cm), **C** IWW obstacles, a 6.6 × 0.9-m walkway instrumented with multiple Microsoft Kinect for Windows sensors and a projector to present two suddenly appearing obstacles (projected red rectangles of 0.4 × 0.9 m) in a gait-dependent (i.e., one obstacle at a predicted foot-placement position appearing two steps ahead) and a position-dependent (i.e., one obstacle at an unpredictable but predefined position appearing when a participant’s ankle was within 2 m from that obstacle) manner. Participants performed those assessments with and without a cognitive dual task

At T0, T1, T2, and T3, participants performed three different walking tasks (see [21, 22] for more details): (1) the standard 10MWT [23], (2) a context-specific 10MWT with stationary physical context (10MWT context), and (3) a context-specific Interactive Walkway assessment with suddenly appearing projected obstacles in a gait-dependent manner (IWW obstacles) to assess walking adaptability under time pressure [24, 25]. All three tasks were performed both with and without the simultaneous performance of a cognitive task, resulting in six walking conditions (Table 1). Tasks were performed in a randomized order. The standard 10MWT (task 1) and 10MWT context (task 2) were both performed three times at a self-selected comfortable walking speed (Fig. 2A,B). The 10MWT context (task 2) comprised three physical obstacles, a tandem-walking path, and three stepping targets. Participants were instructed to step over the obstacles, step onto the targets, and step in-between the tandem-path lines.

**Table 1:** Tasks

	Tasks					
	1) 10MWT		2) 10MWT context		3) IWW obstacles	
	1a) 10MWT	1b) 10MWT + cognitive task	2a) 10MWT context	2b) 10MWT context + cognitive task	3a) IWW obstacles	3b) IWW obstacles + cognitive task
	Outcome measures					
Walking speed	Walking speed (m/s), primary outcome measure	Walking speed (m/s)	(Context-specific) walking speed (m/s)	(Context-specific) walking speed (m/s)	(Context-specific) walking speed (m/s)	(Context-specific) walking speed (m/s)
Walking adaptability			Walking-adaptability score (0–10) Sum of subscores for obstacle avoidance, tandem walking, and targeted stepping^a^	Walking-adaptability score (0–10) Sum of subscores for obstacle avoidance, tandem walking, and targeted stepping ^a^	Walking-adaptability score (0–10) Sum of the points received for the first 10 obstacles ^b^	Walking-adaptability score (0–10) Sum of the points received for the first 10 obstacles ^b^
Cognitive performance		Cognitive performance (the number of correct subtractions per second; n/s)		Cognitive performance (the number of correct subtractions per second; n/s)		Cognitive performance (the number of correct subtractions per second; n/s)
Cognitive-motor interference	Cognitive-motor interference (%) Average of the dual-task effects of walking speed and the cognitive-task performance score (with sitting as single-task reference)		Cognitive-motor interference (%) Average of the dual-task effects of walking speed, the walking-adaptability score, and the cognitive-task performance score (with sitting as single-task reference)		Cognitive-motor interference (%) Average of the dual-task effects of walking speed, the walking-adaptability score, and the cognitive-task performance score (with sitting as single-task reference)	

^a^To be classified as a successfully avoided obstacle, both feet had to stay clear of the obstacle without stepping next to it, circumduction of the hip, or hitting the obstacle (one point per successfully avoided obstacle, with a maximum of three points). For successful targeted stepping, the whole foot had to be placed within the target without allowing intermediate steps (one point per successfully hit target, with a maximum of three points). Because the total number of steps for tandem walking was expected to vary among participants (5.41 ± 1.77 steps, according to the results), we categorized successful tandem walking based on the percentage of correct steps within the narrow-walking path: one point for 0–25% correct steps, two points for 26–50% correct steps, three points for 51–75% correct steps, and four points for 76–100% correct steps

^b^To be classified as a successfully avoided obstacle, both feet had to be placed outside the area of the projected obstacle (i.e., no overlap of shoe and obstacle; one point per obstacle)

The IWW obstacles (task 3) (with and without cognitive task) comprised two suddenly appearing visual obstacles in the form of a projected red rectangle, presented in both a gait-dependent (i.e., at a predicted foot-placement position) and a position-dependent (i.e., at an unpredictable but predefined position) manner. Ten runs were performed, including three dummy trials without obstacles (to retain unpredictability), at a self-selected comfortable walking speed (Fig. 2C). Participants were instructed to step over the suddenly appearing projected obstacle images.

The cognitive dual-task was a serial-3 subtraction task, which had to be performed by counting backwards out loud. The number to start with was varied to avoid task-familiarization. Participants practiced this subtraction task for 30 s while sitting. During all dual-task conditions, participants were instructed to simultaneously perform both tasks as effectively as possible at a self-selected walking speed. Additionally, a 60-s subtraction task was performed while sitting to determine the degree of cognitive-motor interference (i.e., using sitting as the single-task reference for cognitive-task performance, see below). This 60-s seated subtraction task was randomized with the six walking conditions.

### Primary and secondary outcome measures

The primary outcome measure was walking speed (m/s) as determined with the standard 10MWT (averaged over the 3 repetitions as recommended [23]). Secondary outcome measures were measures for context-specific walking speed, walking-adaptability performance, cognitive dual-task performance, cognitive-motor interference, participants’ experience, and amount of walking practice. As specified in Table 1, context-specific walking speed (in m/s) was determined with 10MWT context (averaged over the 3 repetitions) and IWW obstacles trials (averaged over trials, involving the first 10 projected obstacles, excluding dummy trials). Walking-adaptability performance was assessed from the 10MWT context as the sum of subscores obtained for obstacle avoidance, tandem walking, and targeted stepping, averaged over the three repetitions (range 0–10, 1 point per obstacle, 1 point per target, and max 4 points for tandem walking) and from the IWW obstacles as the sum of the points received for the first 10 obstacles to obtain the same scoring range as for the 10MWT context assessment (range 0–10). Walking adaptability was scored manually by two observers through visual inspection of sagittal video recordings and averaged in case of discrepancies. Details regarding the walking-adaptability performance scores can be found in a previous publication (2018) [22] and in Table 1.

Cognitive dual-task performance (the number of correct subtractions per second; sub/s) and cognitive-motor interference (dual-task effects) scores, as determined with the 10MWT with and without context both with and without the cognitive task and IWW obstacles with and without cognitive task [22] (Fig. 2), were again averaged over the 3 repetitions and the trials involving the first 10 projected obstacles excluding dummy trials, respectively. Cognitive-motor interference during dual-task walking was quantified using the average of the respective dual-task effects of walking speed, the walking-adaptability performance score, and the cognitive-task performance (with sitting as single-task reference), that is, motor (walking speed, walking adaptability) and cognitive scores were combined to reflect overall task performance. Following [26], dual-task effects were defined as 100% × (dual-task performance − single-task performance)/single-task performance, with a negative cognitive-motor interference score indicating overall poorer dual-task than single-task performance.

Participants’ experience and attitude towards the interventions were assessed with a purpose-designed evaluation questionnaire consisting of 1–10 rating scales and multiple-choice questions assessing participants’ experience, attitude towards the interventions, improvements, and discomforts during and after training (see Additional file 1: Appendix 1).

The hypothesis of there being different amounts of walking practice per session between CT and FP was tested by comparing the total number of steps and the number of adaptive steps taken per session for two subgroups (CT *n* = 10 and FP *n* = 10). This process measure was obtained using the treadmill’s inbuilt step counter (CT) and by counting the number of steps (FP) using video recordings of a random selection of training sessions by two observers (averaged in case of discrepancies).

### Statistical analysis

Participant characteristics and baseline performance were compared between the two intervention groups using independent *t*-tests for normally distributed interval variables, Mann-Whitney *U* tests for ordinal and non-normal interval variables and Fisher’s exact tests for nominal variables. We used a different statistical analysis (with correction for baseline values) compared to the one described in the study protocol [15], because of the large variation in the baseline (pre-intervention) outcome measures within the groups. We describe “between-groups” and “change-over-time” analyses separately.

### Between-group analyses

For comparing the effects of the interventions on the outcome measures, we calculated per intervention group changes in outcome measures by subtracting baseline values (T0) from the values at each time point (T1, T2, and T3). These change scores of the outcome measures were analyzed using ANCOVA with correction for baseline values. We analyzed ordinal and non-normally distributed variables, notably the participants’ experience and attitude towards the interventions, using Mann-Whitney *U* tests. The amount of walking practice was compared between intervention groups using independent *t*-tests for the total number of steps and the number of adaptive steps taken per training session.

### Change-over-time analyses

To analyze the change over time in the primary outcome measure walking speed and secondary outcome measures context-specific walking speed, walking-adaptability performance, cognitive dual-task performance, and cognitive-motor interference (for all participants, compared to baseline, averaged over groups), we performed paired samples *t*-tests or Wilcoxon signed rank tests for ordinal or non-normally distributed variables at each time point (T1, T2, and T3).

The level of significance was set at *p* < 0.05, while 0.05 < *p* < 0.075 was seen as a tendency towards significance. Effect sizes are presented as partial *ƞ*^*2*^ for ANCOVA or *r* for the other tests. This trial was not an intention-to-treat analysis because dropouts were excluded from the analysis and only complete case data were used.

## Results

**Fig. 3:**
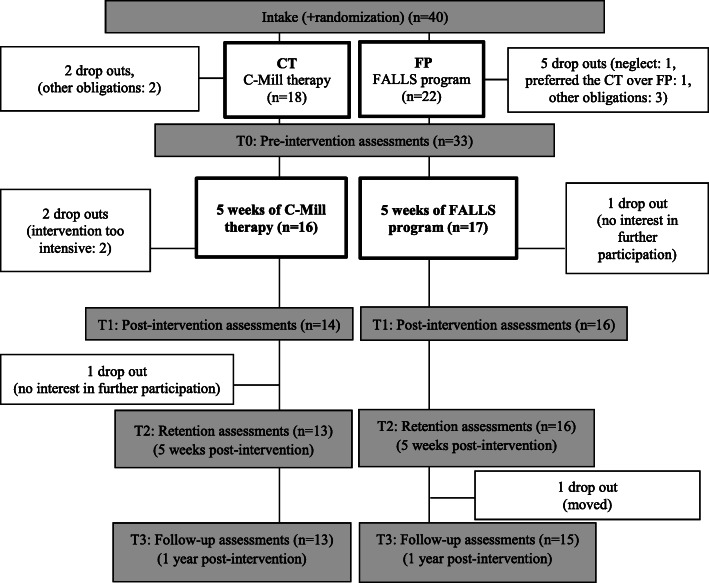
Flow chart with an overview of the procedures and group distribution

Forty participants were recruited and randomized for this trial, out of which 33 participants subsequently performed the pre-intervention tests. Seven participants dropped out mainly due to other obligations (Fig. 3). Thirty of these 33 participants (CT *n* = 14 and FP *n* = 16) completed the intervention and post-intervention assessments (T1), out of which 29 completed post-intervention retention (T2) and 28 post-intervention follow-up (T3). Figure 3 shows the distribution over groups and reasons for dropout. Table 2 provides the characteristics of all participants who completed the pre-intervention assessments (*n* = 33). Participants’ characteristics did not differ significantly between intervention groups.

**Table 2:** Participants’ characteristics

	C-Mill therapy (*n* = 16)	FALLS program (*n* = 17)	*p* value
Gender (female/male)	9/7	5/12	0.13^c^
Age at intake (years)	52 ± 13	59 ± 10	0.13^a^
Height (cm)	171 ± 10	175 ± 7	0.40^a^
Body mass (kg)	76 ± 13	80 ± 14	0.18^a^
Time since stroke at intake (months, median)	46 (6–372)	20 (13–110)	*0.05*^*c*^
Side of lesion (left/right)	9/7	8/9	0.66^c^
Functional Ambulation Category (FAC) at intake (1–5, median)	5 (4–5)	5 (4–5)	0.99^c^
Berg Balance Scale (BBS) (0–56, median)	55 (39–56)	53 (46–56)	0.58^c^
Mini-Mental State Examination MMSE (0–30, median)	29 (26–30)	28 (22–30)	0.63^c^
Assistive device (none/(k)evo/walking cane)	6/4/6	10/3/4	0.22^c^
Level of activity (1–5 days a week at least 30 min moderate active)	1 (0–5)	2 (0–5)	0.31^c^
Falls past year (*n*, %)	8, 50%	13, 76%	0.12^b^
Fear of falling (1 (no fear of falling)–10 (extreme fear of falling))	5 (1–9)	3 (1–10)	0.31^c^
Living situation (independently/independently with help)	3/13	6/11	0.42^c^
Comorbidities (*n*, %)	9, 57%	10, 59%	0.58^b^

*p* values were obtained using ^a^Independent *t*-test, ^b^Fisher’s exact test, or ^c^Mann-Whitney *U* test. Tendencies towards significant differences are presented in italic (0.05 < *p* < 0.075)

### Between-group analyses

#### Walking speed (standard and context-specific)

The CT group showed a significantly greater improvement in walking speed for the 10MWT context than the FP group post-intervention (*p* < 0.05, *ƞ*^*2*^ = 0.17), which was no longer significant at retention and follow-up (Table 3). No other significant differences in standard or context-specific walking speed between groups were found.

**Table 3:** Between-group results (standard and context-specific walking speed)

Outcome (*n*: CT, FP)		CT	FP	Difference between groups (mean difference, *p* value, effect size)
10MWT (m/s)	T0 (14,16)	0.85 ± 0.33	0.91 ± 0.28	− 0.06, 0.59, 0.10^a^
	T1–T0 (14,16)	0.04 ± 0.14	0.05 ± 0.14	− 0.01, 0.81, 0.00^b^
	T2–T0 (13,16)	0.02 ± 0.09	0.03 ± 0.11	− 0.01, 0.72, 0.01^b^
	T3–T0 (13,15)	0.02 ± 0.13	0.06 ± 0.14	− 0.04, 0.31, 0.04^b^
10MWT cognitive (m/s)	T0 (14,16)	0.67 ± 0.25	0.79 ± 0.25	− 0.12, 0.21, 0.24^a^
	T1–T0 (14,16)	0.09 ± 0.14	0.01 ± 0.08	0.08, 0.12, 0.09^b^
	T2–T0 (13,16)	0.06 ± 0.11	0.05 ± 0.10	0.01, 0.96, 0.00^b^
	T3–T0 (13,15)	0.07 ± 0.18	0.03 ± 0.12	0.04, 0.75, 0.00^b^
10MWT context (m/s)	T0 (14,16)	0.56 ± 0.25	0.59 ± 0.17	− 0.03, 0.74, 0.06^a^
	T1–T0 (12,16)*	0.12 ± 0.11	0.03 ± 0.11	**0.09, 0.03, 0.17**^**b**^
	T2–T0 (13,16)	0.14 ± 0.13	0.07 ± 0.13	0.07, 0.10, 0.10^b^
	T3–T0 (13,15)	0.09 ± 0.10	0.08 ± 0.13	0.01, 0.84, 0.00^b^
10MWT context and cognitive (m/s)	T0 (14,16)	0.46 ± 0.24	0.55 ± 0.22	− 0.09, 0.30, 0.19^a^
	T1–T0 (14,16)	0.14 ± 0.13	0.06 ± 0.13	0.08, 0.14, 0.08^b^
	T2–T0 (13,16)	0.17 ± 0.16	0.07 ± 0.12	0.10, 0.14, 0.08^b^
	T3–T0 (13,15)	0.09 ± 0.19	0.07 ± 0.17	0.02, 0.62, 0.01^b^
IWW obstacles (m/s)	T0 (14,14)*	0.69 ± 0.30	0.85 ± 0.18	− 0.16, 0.09, 0.36^a^
	T1–T0 (11,14)*	0.07 ± 0.10	0.03 ± 0.13	0.04, 0.65, 0.01^b^
	T2–T0 (8,13)*	0.08 ± 0.06	0.04 ± 0.13	0.04, 0.64, 0.01^b^
	T3–T0 (5,11)*	0.06 ± 0.05	0.05 ± 0.17	0.01, 0.59, 0.02^b^
IWW obstacles and cognitive (m/s)	T0 (14,14)*	0.61 ± 0.28	0.74 ± 0.15	− 0.14, 0.13, 0.33^a^
	T1–T0 (11,14)*	0.07 ± 0.14	0.05 ± 0.17	0.02, 0.74, 0.01^b^
	T2–T0 (8,13)*	0.09 ± 0.07	0.08 ± 0.12	0.01, 0.96, 0.00^b^
	T3–T0 (5,11)*	0.03 ± 0.03	0.09 ± 0.12	− 0.06, 0.17, 0.14^b^

Mean difference represents the difference between groups (CT-FP) at T0 and mean differences in change values at T1, T2, and T3. *p* values were obtained using ^a^Independent *t*-test or ^b^ANCOVA with baseline performance as covariate. Significant differences are presented in bold (*p* < 0.05) and tendencies are presented in italic (0.05 < *p* < 0.075). *Some participants were excluded (see degrees of freedom) due to technical problems with the IWW and/or video footage

#### Walking adaptability

No significant between-group differences were found (Table 4).

**Table 4:** Between-groups results (walking adaptability)

Outcome	(*n*: CT, FP)	CT	FP	Difference between groups (mean difference, *p* value, effect size)
10MWT context (1–10)	T0 (14,16)	5.59 ± 2.43	4.97 ± 2.21	0.62, 0.47, 0.09^a^
	T1–T0 (12,16)*	0.49 ± 1.03	0.27 ± 1.79	0.22, 0.58, 0.01^b^
	T2–T0 (10,16)*	0.26 ± 1.53	0.23 ± 1.94	0.03, 0.48, 0.02^b^
	T3–T0 (7,15)*	0.37 ± 1.68	0.07 ± 2.70	0.30, 0.69, 0.00^b^
10MWT context with cognitive task (1–10)	T0 (14,16)	5.31 ± 2.29	4.36 ± 2.03	0.95, 0.24, 0.22^a^
	T1–T0 (13,16)*	0.42 ± 1.19	0.16 ± 1.33	0.26, 0.45, 0.02^b^
	T2–T0 (10,16)*	− 0.38 ± 1.15	0.34 ± 1.93	− 0.72, 0.20, 0.07^b^
	T3–T0 (7,15)*	0.17 ± 1.91	0.28 ± 2.53	− 0.11, 0.81, 0.00^b^
IWW obstacles (1–10)	T0 (14,14)*	5.96 ± 3.52	6.25 ± 3.51	− 0.29, 0.83, 0.04^a^
	T1–T0 (11,14)*	3.09 ± 2.56	2.11 ± 2.31	0.98, 0.59, 0.01^b^
	T2–T0 (8,13)*	2.81 ± 2.55	1.58 ± 2.24	0.60, 0.79, 0.00^b^
	T3–T0 (6,10)*	3.42 ± 3.68	0.9 ± 3.06	2.52, 0.93, 0.00^b^
IWW obstacles with cognitive task (1–10)	T0 (14,14)*	4.75 ± 3.71	5.21 ± 2.96	− 0.46, 0.72, 0.07^a^
	T1–T0 (11,14)*	1.95 ± 1.67	0.82 ± 2.25	1.13, 0.25, 0.06^b^
	T2–T0 (8,13)*	3.50 ± 2.33	1.58 ± 1.30	1.92, 0.11, 0.14^b^
	T3–T0 (6,10)*	1.42 ± 2.71	1.05 ± 3.55	0.37, 0.28, 0.09^b^

Mean difference represents the difference between groups (CT–FP) at T0 and mean differences in change values at T1, T2, and T3. *p* values were obtained using ^a^Independent *t*-test or ^b^ANCOVA with baseline performance as covariate. *Some participants were excluded (see degrees of freedom) due to technical problems with the IWW and/or video footage

#### Cognitive dual-task performance and cognitive-motor interference

Post-intervention (i.e., at T1), the CT group showed a tendency to a greater improvement in cognitive performance during the standard 10MWT with cognitive dual-task compared to the FP group (*p* = 0.06, *ƞ*^*2*^ = 0.13), which disappeared at retention and follow-up (Table 5). Cognitive-motor interference outcomes did not differ significantly between groups (Table 5).

**Table 5:** Between-group results (cognitive dual-task performance and cognitive-motor interference)

Outcome	(*n*: CT, FP)	CT	FP	Difference between groups (mean difference, *p* value, effect size)
10MWT cognitive (sub/s)	T0 (14,16)	0.43 ± 0.12	0.51 ± 0.31	− 0.08, 0.36, 0.21^a^
	T1–T0 (14,16)	0.09 ± 0.10	0.02 ± 0.09	*0.07, 0.06, 0.13*^*b*^
	T2–T0 (12,16)*	0.09 ± 0.07	0.05 ± 0.14	0.04, 0.59, 0.01^b^
	T3–T0 (8,14)*	0.10 ± 0.08	0.04 ± 0.19	0.06, 0.88, 0.00^b^
10MWT context and cognitive (sub/s)	T0 (14,16)	0.31 ± 0.15	0.37 ± 0.25	− 0.07, 0.36, 0.18^a^
	T1–T0 (14,16)	0.11 ± 0.11	0.06 ± 0.12	0.05, 0.28, 0.04^b^
	T2–T0 (12,16)*	0.08 ± 0.08	0.05 ± 0.08	0.03, 0.33, 0.04^b^
	T3–T0 (8,15)*	0.06 ± 0.07	0.03 ± 0.18	0.03, 0.59, 0.01^b^
IWW obstacles and cognitive (sub/s)	T0 (14,14)*	0.33 ± 0.12	0.51 ± 0.31	− *0.19, 0.05, 0.46*^*a*^
	T1–T0 (11,14)*	0.15 ± 0.14	0.12 ± 0.15	0.03, 0.40, 0.03 ^b^
	T2–T0 (8,13)*	0.02 ± 0.08	0.04 ± 0.14	− 0.02, 0.58, 0.02^b^
	T3–T0 (5,11)*	0.05 ± 0.14	− 0.22 ± 0.30	0.27, 0.16, 0.13^b^
Cognitive-motor interference 10MWT (%)	T0 (14,16)	− 6 (− 28 to − 1)	− 1(− 40–52)	− **9, 0.03, 0.39**^**c**^
	T1–T0 (14,16)	3 ± 22	− 5 ± 20	8, 0.83, 0.00^b^
	T2–T0 (12,16)*	3 ± 14	2 ± 22	1, 0.58, 0.01^b^
	T3–T0 (8,14)*	34 ± 84	13 ± 26	21, 0.55, 0.02^b^
Cognitive-motor interference 10MWT context (%)	T0 (14,16)	− 15 (− 45–23)	− 9 (− 36–5)	− 2, 0.48, 0.13^c^
	T1–T0 (12,16)*	− 1 ± 15	2 ± 18	− 3, 0.46, 0.02^b^
	T2–T0 (10,16)*	2 ± 24	7 ± 21	− 5, 0.50, 0.02^b^
	T3–T0 (7,15)*	− 9 ± 17	8 ± 19	− 17, 0.11, 0.13^b^
Cognitive-motor interference IWW obstacles (%)	T0 (14,14)*	− 17 (− 39–20)	8 (− 32–223)	− **25, 0.04, 0.38**^**c**^
	T1–T0 (11,14)*	0 ± 21	− 22 ± 69	22, 0.76, 0.00 ^b^
	T2–T0 (8,13)*	6 ± 27	− 20 ± 68	26, 0.62, 0.01^b^
	T3–T0 (5,10)*	− 14 ± 22	− 33 ± 78	19, 0.98, 0.00^b^

Mean difference represents the difference between groups (CT-FP) at T0 and mean differences in change values at T1, T2, and T3. *p* values were obtained using ^a^Independent *t-*test, ^b^ANCOVA with baseline performance as covariate, or ^c^Mann-Whitney *U* test. Significant differences are presented in bold (*p* < 0.05) and tendencies are presented in italic (0.05 < *p* < 0.075). *Some participants were excluded (see degrees of freedom) due to technical problems with the IWW and/or video footage

#### Participants’ experience and attitude towards the interventions

Participants scored the interventions as useful, motivating, fun, challenging, enjoyable, and suitable; they were initially somewhat reserved but would recommend the intervention they underwent to peers (Additional file 2: Appendix 2.1). Participants of the CT group showed a tendency to higher ratings on “recommend it to peers” and “perceived fun” compared to participants of the FP group (*U* = 69.5, *p* = 0.07, *r* = 0.34, *U* = 70.0, *p* = 0.07, *r* = 0.33, respectively). After both interventions, participants described an increase in physical fitness, safety of walking, walking speed, and confidence during walking (Additional file 2: Appendix 2.2). In the CT group, significantly more participants reported an increase in physical fitness compared to the FP group (*U* = 65.0, *p* < 0.05, *r* = 0.43). The CT group also perceived significantly more discomforts during the training than the FP group (*U* = 62.5, *p* < 0.05, *r* = 0.42). However, these were only mild, and no significant difference was found in the perceived discomforts after training (Additional file 2: Appendix 3.2).

#### Amount of walking practice

About twice as many steps and adaptive steps were taken during CT training (2779 ± 582, 2405 ± 505 steps, respectively) than during FP training (1464 ± 196, 1130 ± 175 steps, respectively). Both the total number of steps per session of equal duration (*t*(18) = − 6.88, *p* < 0.01, *r* = 0.85) and the number of adaptive steps per session of equal duration (*t*(18) = − 7.54, *p* < 0.01, *r* = 0.87) were significantly higher in the CT group.

### Change-over-time analyses

#### Walking speed (standard and context-specific)

Whereas walking speed of the standard 10MWT did not change significantly over time, significant (or near significant) improvements in context-specific walking speed were observed post-intervention, at retention and follow-up for the participants of both groups combined (Table 6).

**Table 6:** Change-over-time results (standard and context-specific walking speed)

Outcome	(*n*: CT, FP)	CT and FP	Change over time, both groups (mean difference, *p* value, effect size)
10MWT (m/s)	T0 (14,16)	0.88 ± 0.30	–
	T1 (14,16)	0.92 ± 0.30	0.04, 0.10, 0.30
	T2 (13,16)	0.91 ± 0.30	0.03, 0.12, 0.30
	T3 (13,15)	0.93 ± 0.31	0.05, 0.13, 0.29
10MWT cognitive (m/s)	T0 (14,16)	0.73 ± 0.25	–
	T1 (14,16)	0.78 ± 0.25	**0.05, 0.04, 0.38**
	T2 (13,16)	0.78 ± 0.26	**0.05, 0.01, 0.50**
	T3 (13,15)	0.77 ± 0.27	0.04, 0.13, 0.29
10MWT context (m/s)	T0 (14,16)	0.57 ± 0.21	–
	T1 (12,16)*	0.64 ± 0.26	**0.07, 0.00, 0.53**
	T2 (13,16)	0.66 ± 0.26	**0.09, 0.00, 0.61**
	T3 (13,15)	0.64 ± 0.21	**0.07, 0.00, 0.64**
10MWT context and cognitive (m/s)	T0 (14,16)	0.51 ± 0.23	–
	T1 (14,16)	0.61 ± 0.26	**0.10, 0.00, 0.59**
	T2 (13,16)	0.62 ± 0.23	**0.11, 0.00, 0.64**
	T3 (13,15)	0.58 ± 0.21	**0.07, 0.03, 0.40**
IWW obstacles (m/s)	T0 (14,14)*	0.77 ± 0.25	–
	T1 (11,14)*	0.82 ± 0.25	**0.05, 0.04, 0.41**
	T2 (8,13)*	0.82 ± 0.26	**0.05, 0.03, 0.46**
	T3 (5,11)*	0.84 ± 0.28	0.07, 0.14, 0.14
IWW obstacles and cognitive (m/s)	T0 (14,14)*	0.67 ± 0.23	–
	T1 (11,14)*	0.72 ± 0.21	**0.05, 0.02, 0.45**
	T2 (8,13)*	0.75 ± 0.23	**0.08, 0.00, 0.64**
	T3 (5,11)*	0.76 ± 0.25	**0.09, 0.02, 0.33**

Mean difference represents the difference over time at T1, T2, and T3 compared to baseline (T0). *p* values were obtained using Paired samples *t*-tests. Significant differences are presented in bold (*p* < 0.05). *Some participants were excluded (see degrees of freedom) due to technical problems with the IWW and/or video footage

#### Walking adaptability

Walking adaptability improved over time, albeit only for walking-adaptability outcomes assessed with IWW obstacles and IWW obstacles with the cognitive task post-intervention, at retention and follow-up for the participants of both groups combined (Table 7).

**Table 7:** Change-over-time results (walking adaptability)

Outcome	(*n*: CT, FP)	CT and FP	Change over time, both groups (mean difference, *p* value, effect size)
10MWT context (1–10)	T0 (14,16)	5.26 ± 2.30	–
	T1 (12,16)	5.46 ± 2.15	0.20, 0.20, 0.24
	T2 (10,16)*	5.01 ± 2.34	− 0.15, 0.91, 0.02
	T3 (7,15)*	5.38 ± 2.48	0.12, 0.76, 0.00
10MWT context with cognitive task (1–10)	T0 (14,16)	4.80 ± 2.17	–
	T1 (13,16)*	4.99 ± 2.28	0.19, 0.24, 0.22
	T2 (10,16)*	4.55 ± 1.98	− 0.25, 0.81, 0.05
	T3 (7,15)*	4.89 ± 2.47	0.09, 0.62, 0.01
IWW obstacles (1–10)	T0 (14,14)*	6.11 ± 3.45	–
	T1 (11,14)*	8.26 ± 2.40	**2.15, 0.00, 0.73**
	T2 (8,13)*	7.74 ± 2.70	**1.63, 0.00, 0.66**
	T3 (6,10)*	7.50 ± 2.08	*1.39, 0.05, 0.66*
IWW obstacles with cognitive task (1–10)	T0 (14,14)*	4.98 ± 3.30	–
	T1 (11,14)*	6.08 ± 3.27	**1.10, 0.00, 0.55**
	T2 (8,13)*	6.90 ± 2.80	**1.92, 0.00, 0.24**
	T3 (6,10)*	5.75 ± 3.16	0.77, 0.16, 0.13

Mean difference represents the difference over time at T1, T2, and T3 compared to baseline (T0). *p* values were obtained using Paired samples *t*-tests. Significant differences are presented in bold (*p* < 0.05) and tendencies are presented in italic (0.05 < *p* < 0.075). *Some participants were excluded (see degrees of freedom) due to technical problems with the IWW and/or video footage

#### Cognitive dual-task performance and cognitive-motor interference

Cognitive-motor interference outcomes did not differ significantly over time for the participants of both groups combined (Table 8).

**Table 8:** Change-over-time results (cognitive dual-task performance and cognitive-motor interference)

Outcome	(*n*: CT, FP)	CT and FP	Change over time, both groups (mean difference, *p* value, effect size)
10MWT cognitive (sub/s)	T0 (14,16)	0.47 ± 0.24	–
	T1 (14,16)	0.52 ± 0.24	**0.05, 0.01, 0.49**
	T2 (12,16)*	0.54 ± 0.24	**0.07, 0.00, 0.52**
	T3 (8,14)*	0.61 ± 0.33	0.14, 0.10, 0.12
10MWT context and cognitive (sub/s)	T0 (14,16)	0.34 ± 0.21	–
	T1 (14,16)	0.42 ± 0.23	**0.08, 0.00, 0.56**
	T2 (12,16)*	0.41 ± 0.18	**0.07, 0.00, 0.61**
	T3 (8,15)*	0.39 ± 0.16	0.05, 0.21, 0.07
IWW obstacles and cognitive (sub/s)	T0 (14,14)*	0.42 ± 0.25	–
	T1 (11,14)*	0.56 ± 0.31	**0.14, 0.00, 0.69**
	T2 (8,13)*	0.42 ± 0.19	0.00, 0.47, 0.16
	T3 (5,11)*	0.35 ± 0.14	− 0.7, 0.09, 0.18
Cognitive-motor interference 10MWT (%)	T0 (14,16)	− 4 (− 40–52)	–
	T1 (14,16)	− 6 ± 18	− 2, 0.72, 0.07
	T2 (12,16)*	− 2 ± 18	2, 0.55, 0.12
	T3 (8,14)*	16 ± 53	20, 0.08, 0.14
Cognitive-motor interference 10MWT context (%)	T0 (14,16)	− 13 (− 45–23)	–
	T1 (12,16)*	− 12 ± 14	1, 0.81, 0.05
	T2 (10,16)*	− 7 ± 19	6, 0.25, 0.23
	T3 (7,15)*	−8 ± 13	5, 0.51, 0.02
Cognitive-motor interference IWW obstacles (%)	T0 (14,14)*	− 4 (− 39–223)	–
	T1 (11,14)*	− 14 ± 15	− 10, 0.26, 0.23
	T2 (8,13)*	− 10 ± 19	− 6, 0.44, 0.17
	T3 (5,10)*	− 21 ± 16	− 17, 0.13, 0.15

Mean difference represents the difference over time at T1, T2, and T3 compared to baseline (T0). *p* values were obtained using Paired samples *t-*test. Significant differences are presented in bold (*p* < 0.05) and tendencies are presented in italic (0.05 < *p* < 0.075). *Some participants were excluded (see degrees of freedom) due to technical problems with the IWW and/or video footage

## Discussion

This study compared the efficacy of a novel treadmill-based C-Mill therapy with that of the standard overground FALLS program in a cohort of persons minimally 3 months after stroke in the Netherlands. By and large, this study showed neutral between-group findings. No significant differences between the two intervention groups were found in the primary outcome measure standard walking speed. But for the secondary outcome measures, we observed a greater improvement in context-specific walking speed with stationary physical context of the C-Mill therapy compared to the FALLS program post-intervention, which was no longer significant at retention. Both interventions were well received, but C-Mill therapy scored significantly better on perceived increased fitness (based on the evaluation questionnaire), which may be attributed to the significantly higher amount of walking practice (twice as many steps during CT than FP training sessions of equal duration).

Both walking-adaptability interventions yielded a very high proportion of adaptive steps. As expected, C-Mill therapy showed a greater amount of walking practice. About twice as many steps (total number of steps and number of adaptive steps) were taken during a CT training session compared to a FP training session of equal duration. This may have positively influenced perceived fitness and possibly context-specific walking speed but does not readily explain other between-group differences (or the absence thereof). Other differences between the interventions could have played a role, such as the type of context (stationary physical context vs. suddenly appearing projector-generated context). Whereas training with physical context more closely mimics walking in daily life with physical obstacles, projector-generated context reduces the actual physical risk of falling during the training. As a result, the consequence of failure in walking-adaptability performance was greater in the FALLS program than in C-Mill therapy. Future training interventions should therefore take the type of context into account, as this could potentially result in a difference in task prioritization [22].

Differences in amount of walking practice and context notwithstanding, both interventions of this essentially neutral between-group RCT showed significant long-term effects (1-year follow-up), which have not yet been demonstrated before. The significant improvements over time for both interventions combined suggest that both interventions contain important aspects for improving walking speed and walking adaptability. Particularly, our participants showed significant improvements in context-specific walking speed, walking-adaptability performance (but only when assessed with the IWW), and cognitive dual-task performance immediately post-intervention, at retention and follow-up, but, in contrast to a recent proof-of-concept study on walking-adaptability therapy [12], not so for our primary outcome measure walking speed as determined with the standard 10MWT. The standard 10MWT might not be sensitive or specific enough to evaluate the effect of walking-adaptability training, which highlights the importance of including context-specific walking speed assessments, for which many significant improvements over time were observed (and even minor between-group differences in the change scores; Table 2). Interestingly, the observed improvements in walking-adaptability over time were only significant for the IWW assessment with suddenly appearing projected obstacles and not for the assessments with stationary physical context (Table 3). This finding underscores the importance of assessing step adjustments under high time-pressure demands, as this is especially difficult for people after stroke [3, 8].

These sustained effects of both walking-adaptability interventions in this essentially neutral between-group trial may allow clinicians to opt for either of both forms of training when considering walking-adaptability training for their patients. They may opt for C-Mill therapy in view of the higher amount of walking practice inherent to treadmill walking, or for the FALLS program because of the more realistic overground practice environment with, e.g., physical obstacles and the practice of fall techniques, or for a combination of both interventions. In considering these options, they should keep in mind that C-Mill therapy might require a familiarization period of walking on a treadmill, while the FALLS program is lacking adaptability tasks under high time-pressure demands as well as feedback on performance.

### Limitations and recommendations

Even though the number of participants was established with a power analysis and still sufficient after dropouts, the dropout rate after intake was high (25%) (Fig. 3). A larger (multicenter) trial should be performed to underpin the efficacy of the walking-adaptability interventions, which could benefit from the recently validated development of an automatized, progressive patient-tailored C-Mill training program [27]. Another limitation of this study was the lack of a control group (e.g., no intervention, other usual care than the FALLS program), which prohibits ascribing the observed effects completely to the interventions. Finally, due to the nature of the intervention the assessors, neither physical therapists nor participants were blind to group allocation.

### Conclusion

In a cohort of stroke patients minimally 3 months post stroke, this study showed no between-group differences between a novel treadmill-based C-Mill therapy and a standard overground FALLS program with respect to the primary outcome measure standard walking speed. However, the analyses of the secondary outcome measures suggested that the greater amount of walking practice observed for the C-Mill group, an essential aspect of effective intervention programs after stroke, may underlie the reported increased perceived fitness and observed increased context-specific walking speed for the C-Mill group directly after the intervention. Furthermore, the “change-over-time” results showed that participants of both intervention combined did not show an improvement in the standard walking speed, yet context-specific walking speed and walking adaptability did improve and sustain after the interventions, testifying to the importance of including walking-adaptability training and assessment in rehabilitation post stroke.

## Supplementary Information

**Additional file 1..** Purpose-designed evaluation questionnaire.

**Additional file 2..** Results of the evaluation questionnaire for the treadmill-based C-Mill therapy (CT) and overground FALLS program (FP).

## Data Availability

The datasets used and/or analyzed during the current study are available from the corresponding author on reasonable request.
