# Supplementary material for: Walking-adaptability therapy after stroke: results of a randomized controlled trial
Source: Trials. 2021 Dec 15;22:923. doi: 10.1186/s13063-021-05742-3 (PMC8672482; doi:10.1186/s13063-021-05742-3)
Supplement: Supplementary file 1 — Additional file 1.. Purpose-designed evaluation questionnaire. [file 13063_2021_5742_MOESM1_ESM.docx]

**Appendix 1. Purpose-designed evaluation questionnaire**

| 1  **NO** | 2 | 3 | 4 | 5 | 6 | 7 | 8 | 9 | 10  **YES** |
| --- | --- | --- | --- | --- | --- | --- | --- | --- | --- |

1. Did you perceive the training (C-Mill therapy or FALLS program) as useful? Select the box (mark) that fits best.
2. Did you perceive the training as motivating? Select the box (mark) that fits best.

| 1  **NO** | 2 | 3 | 4 | 5 | 6 | 7 | 8 | 9  **YES** | 10 |
| --- | --- | --- | --- | --- | --- | --- | --- | --- | --- |

1. Did you perceive the training as fun? Select the box (mark) that fits best.

| 1  **NO** | 2 | 3 | 4 | 5 | 6 | 7 | 8 | 9 | 10  **YES** |
| --- | --- | --- | --- | --- | --- | --- | --- | --- | --- |

1. Did you perceive the training as challenging? Select the box (mark) that fits best.

**NO**

| 1  **NO** | 2 | 3 | 4 | 5 | 6 | 7 | 8 | 9 | 10  **YES** |
| --- | --- | --- | --- | --- | --- | --- | --- | --- | --- |

1. Did you perceive the training as enjoyable? Select the box (mark) that fits best.

| 1  **NO** | 2 | 3 | 4 | 5 | 6 | 7 | 8 | 9 | 10  **YES** |
| --- | --- | --- | --- | --- | --- | --- | --- | --- | --- |

| 1 | 2 | 3 | 4 | 5 | 6 | 7 | 8 | 9 | 10 |
| --- | --- | --- | --- | --- | --- | --- | --- | --- | --- |

1. Were you initially cautious about the training? Select the box (mark) that fits best.

**NO**

**YES**

1. Did you perceive the training as suitable after a stroke? Select the box (mark) that fits best.

| 1  **NO**  **YES** | 2 | 3 | 4 | 5 | 6 | 7 | 8 | 9 | 10 |
| --- | --- | --- | --- | --- | --- | --- | --- | --- | --- |

| 1  **NO** | 2 | 3 | 4 | 5 | 6 | 7 | 8 | 9  **YES** | 10 |
| --- | --- | --- | --- | --- | --- | --- | --- | --- | --- |

1. Would you recommend the training to peers? Select the box (mark) that fits best.

*FP only*

1. *Which part of the training did you perceive as easy? ^(multiple answers are possible)^*

**NO**

- *Obstacle course, namely ____________________________*
- *Practicing falling techniques*
- *Walking exercises*

1. *Which part of the training did you perceive as difficult? ^(multiple answers are possible)^*

**NO**

- *Obstacle course, namely ____________________________*
- *Practicing falling techniques*
- *Walking exercises*

*CT only*

1. *Which part of the training did you perceive as easy? ^(multiple answers are possible)^*

**NO**

- *Obstacle avoidance*
- *Goal-directed stepping*
- *Goal-directed stepping with obstacles*
- *Speed adaptations*
- *Walking with tandem steps*
- *The walking-adaptability game*

1. *Which part of the training did you perceive as difficult? ^(multiple answers are possible)^*

- *Obstacle avoidance*
- *Goal-directed stepping*
- *Goal-directed stepping with obstacles*
- *Speed adaptations*
- *Walking with tandem steps*
- *The walking-adaptability game*

1. Did you perceive discomfort(s) DURING one or more training sessions (e.g. severe fatigue, muscle soreness, dizziness, shortness of breath)?

- Yes, multiple times
- Yes, once
- No

If so, what were these discomforts? ^(multiple answers are possible)^:

- Severe fatigue
- Muscle soreness
- Dizziness
- Nausea
- Shortness of breath
- Painful joints, namely __________________________________
- Other, namely____________________________________________

1. Did you perceive discomfort(s) AFTER one or more training sessions (e.g. severe fatigue, muscle soreness, dizziness, shortness of breath)?

- Yes, multiple times
- Yes, once
- No

If so, what were these discomforts? ^(multiple answers are possible)^:

- Severe fatigue
- Muscle soreness
- Dizziness
- Nausea
- Shortness of breath
- Painful joints, namely __________________________________
- Other, namely____________________________________________

1. Did you unintentionally fall during one of the training sessions?

- Yes, multiple times, namely________________________________
- Yes, once
- No

1. What did you think of the duration of a training sessions?

- Good
- Too long
- Too short
- Other, namely__________________________________________

1. What did you think of intensity of the training?

- Good
- Too ligth
- Too hard
- Other, namely__________________________________________

1. Do you think your physical fitness has increased?

- Yes
- No

1. Do you think you have increased your safety during walking in your own environment?

- Yes, continue to question b
- No

b. Can you describe how?

1. Do you think you have increased your walking speed?

- Yes
- No

1. Do you think you have increased your trust during walking?
   - - Yes, continue to question b
     - No

b. If so, where? ^(multiple answers are possible)^

- Indoors
- Outdoors, smooth terrain
- Outdoors, uneven terrain
- Outdoors, busy environment
- Other, namely__________________________________________
