# Supplementary material for: Walking-adaptability therapy after stroke: results of a randomized controlled trial
Source: Trials. 2021 Dec 15;22:923. doi: 10.1186/s13063-021-05742-3 (PMC8672482; doi:10.1186/s13063-021-05742-3)
Supplement: Supplementary file 2 — Additional file 2.. Results of the evaluation questionnaire for the treadmill-based C-Mill therapy (CT) and overground FALLS program (FP). [file 13063_2021_5742_MOESM2_ESM.docx]

**Appendix 2.1. Results of the evaluation questionnaire for the treadmill-based C-Mill therapy (CT) and overground FALLS program (FP)**

FP

CT

|  | **1** | **2** | **3** | **4** | **5** | **6** | **7** | | **8** | **9** | **10** |  | **Median** | ***p*-value, effect size** |
| --- | --- | --- | --- | --- | --- | --- | --- | --- | --- | --- | --- | --- | --- | --- |
| Not useful |  |  |  |  |  |  | 3 | | 5 | 3 | 3 | Useful | 8 | 0.09, 0.31 |
|  |  | 1 |  |  | 2 | 1 | 4 | | 4 | 2 | 2 |  | 8 |  |
|  |  | | | | | | | | | | | | | |
| Not motivating |  |  |  |  |  |  | 3 | | 4 | 6 | 1 | Motivating | 8 | 0.08, 0.32 |
|  |  | 1 |  |  | 2 |  | 3 | | 8 |  | 2 |  | 9 |  |
|  |  | | | | | | | | | | | | | |
| Not fun |  |  |  |  |  |  | 2 | | 7 | 2 | 3 | Fun | 8 | *0.07, 0.33* |
|  |  |  | 1 |  |  | 4 | 2 | | 6 | 1 | 2 |  | 8 |  |
|  | | | | | | | | | | | | |  |  |
| Not challenging |  |  |  |  | 3 | 1 | 2 | | 3 | 4 | 1 | Challenging | 8 | 0.72, 0.07 |
|  |  |  |  | 1 |  | 3 | 4 | | 5 | 2 | 1 |  | 8 |  |
|  | | | | | | | | | | | | |  |  |
| Not enjoyable |  |  |  |  |  |  | 3 | | 5 | 2 | 4 | Enjoyable | 8 | 0.18, 0.24 |
|  |  | 1 |  |  |  | 1 | 4 | | 6 | 2 | 2 |  | 8 |  |
|  | |  | | | | | |  | | | | | | |
| Not suitable |  |  |  | 2 |  |  | 1 | | 5 | 1 | 5 | Suitable | 8 | 0.35, 0.17 |
|  |  |  |  |  | 2 |  | 5 | | 3 | 4 | 2 |  | 8 |  |
|  | | | | | | | | | | | | |  |  |
| Would not recommend it to peers |  |  |  |  |  |  | 3 | | 2 | 5 | 4 | Would recommend it to peers | 8 | *0.07, 0.34* |
|  | 1 |  |  |  | 2 | 1 | 3 | | 2 | 6 | 1 |  | 9 |  |
|  |  |  |  |  |  |  |  | |  |  |  |  |  |  |
| Initially reserved | 4 | 2 | 3 | 1 | 1 |  |  | | 1 | 1 | 1 | Initially not reserved | 5 | 0.51, 0.12 |
|  | 4 | 2 | 1 |  | 2 | 1 | 2 | | 2 | 2 |  |  | 3 |  |

*p*-values were obtained using Mann-Whitney *U*-tests. Significant differences are presented in bold (*p*<0.05) and tendencies are presented in italic (0.05<*p*<0.075).

**Appendix 2.2. Results of the evaluation questionnaire for the treadmill-based C-Mill therapy (CT) and overground FALLS program (FP)**

|  | **C-Mill therapy**  **(n=14)** | **FALLS program**  **(n=16)** | ***p*-value, effect size** |
| --- | --- | --- | --- |
| Perceived discomforts during training sessions | | | |
| No, n (%) | 5 (35.7) | 12 (75.0) | **0.02, 0.42** |
| Yes once, n (%) | 4 (28.6) | 3 (18.8) |  |
| Yes frequently, n (%) | 5 (35.7) | 1 (6.3) |  |
| Perceived discomforts after training sessions | | | |
| No, n (%) | 7 (50.5) | 12 (75.0) | 0.20, 0.23 |
| Yes once, n (%) | 1 (7.1) | 0 (0.0) |  |
| Yes frequently, n (%) | 6 (42.9) | 4 (25.0) |  |
| Increase in | | | |
| Physical fitness, n (%) | 12 (85.7) | 7 (43.8) | **0.02, 0.43** |
| Safety of walking, n (%) | 11 (78.6) | 11 (68.6) | 0.55, 0.11 |
| Walking speed, n (%) | 12 (85.7) | 9 (56.3) | 0.08, 0.32 |
| Confidence during walking, n (%) | 12 (85.7) | 12 (75.0) | 0.47, 0.13 |

*p-*values were obtained using Mann-Whitney *U*-tests. Significant differences are presented in bold (*p*<0.05).
